# Methylation of *HIF3A *promoter CpG islands contributes to insulin resistance in gestational diabetes mellitus

**DOI:** 10.1002/mgg3.583

**Published:** 2019-02-11

**Authors:** Yinghong Zhang, Yangyang Chen, Hongmei Qu, Yuanli Wang

**Affiliations:** ^1^ Department of Obstetrics the Affiliated Yantai Yuhuangding Hospital of Qingdao University Yantai China

**Keywords:** gestational diabetes mellitus (GDM), *HIF3A*, methylation

## Abstract

**Background:**

Gestational diabetes mellitus (GDM) is defined as any degree of glucose intolerance during pregnancy, and will lead to high risk of diabetes even after pregnancy. Hypoxia‐inducible factor (HIF) family proteins are transcriptional factors that are highly correlated with methylation, which might be involved in the regulation of GDM.

**Methods:**

Baseline clinical characteristics of the GDM patients and healthy women were analyzed. Omental tissue from GDM patients and control groups were collected and detected for the expression levels of *HIF1A*, *HIF2A,* and *HIF3A*. The CpG islands of *HIF3A* promoter were predicted by “methprimer” software, and the methylation level of CpG islands was detected by bisulfite sequencing PCR.

**Results:**

*HIF3A* was downregulated in the omental tissue from GDM patients, whereas *HIF1A* and *HIF2A* were not affected. Furthermore, *HIF3A* expression was positively correlated with levels of estrogen receptor α (*ESR1*) and solute carrier family 2 member 4 (*SLC2A4*). Moreover, CpG islands of *HIF3A* promoter were highly methylated in GDM patients. In addition, methylation level of CpG islands could be upregulated by Estradiol (E2) treatment, since high dose of E2 reduced *HIF3A *mRNA expression in 3T3‐L1 adipocytes.

**Conclusion:**

Our findings demonstrate that the expression level of *HIF3A*, but not *HIF1A* or *HIF2A*, is downregulated in GDM patients. The methylation status of *HIF3A* promoter region is highly correlated with GDM, which could be a novel therapeutic target for GDM treatment.

## 1 INTRODUCTION

Gestational diabetes mellitus (GDM) is a condition in which women without diabetes have a high blood glucose level during their pregnancy. GDM affects women worldwide, and approximately 80% of diabetic pregnant women are affected by GDM based on clinical diagnosis (Chiefari, Arcidiacono, Arcidiacono, Foti, & Brunetti, 2017). Unlike type I diabetes, GDM is not caused by insufficient source of insulin, but by insulin resistance (Li et al., 2018). During pregnancy, massive secretion of various hormones, such as estrogen, cortisol, progesterone, placental lactogen, thyroxin and growth hormone, interferes with the binding of insulin to its receptor, resulting in poor efficacy of insulin (Shi et al., 2014). For example, unconjugated estriol was significantly correlated with increased risk of GDM (Hur, Cho, Cho, Baek, & Lee, 2017; Montelongo, Lasuncion, Lasuncion, Pallardo, & Herrera, 1992; Qi et al., 2017). Thus, pregnant women need more insulin to reduce their blood sugar level. GDM can cause obesity, type II diabetes (T2D), cardiovascular disease and chronic hypertension during pregnancy. The newborns might also be at increased risk of excessive birth weight, hypoglycemia, respiratory distress syndrome, intellectual disability and the chance of developing T2D later in life (Nankervis, Price, Price, & Conn, 2018). Therefore, studying the molecular mechanisms underlying GDM pathogenesis is critical to develop diagnostic and therapeutic strategies.

DNA methylation is a procedure by which methyl groups are added to DNA molecules. It usually occurs at the gene promoter region, such as a CpG island (Dick et al., 2014; Huang et al., 2018). A CpG island is defined as a region containing 300–3,000 base pairs with a C + G content more than 0.6. DNA methylation has an important role in regulating gene activation without changing gene sequence, along with ubiquitination, acetylation and phosphorylation (Moore, Le, Le, & Fan, 2013). An increasing number of evidences indicate that deregulation of DNA methylation is involved in pathogenesis of GDM (Shen et al., 2007). It has been reported that global placental DNA hyper‐methylation is associated with GDM (Kang, Lee, Li, Hsu, & Lin, 2017).

Hypoxia‐inducible factor‐3α (HIF3A, OMIM association number, 609,976) is a member of transcriptional factor family of hypoxia‐inducible factors (HIFs). HIF family regulates a wide range of target genes related to adipose tissue dysfunction, inflammation, and cancer (Makino et al., 2001). HIFs are heterologous dimers composed of an oxygen‐labile α‐subunit and a constitutively expressed β‐subunit. The three types of the α‐subunits, HIF1A (OMIM association number, 603,348), HIF2A (OMIM association number, 603,349), or HIF3A, dimerize with hypoxia‐inducible factor‐1β to form heterodimers to exert different functions (Hara, Hamada, Hamada, Kobayashi, Kondo, & Imura, 2001). Recent studies have shown that the methylation of *HIF3A* is associated with the metabolism in adipose tissue and insulin sensitivity, which are two major factors contributing to GDM development (Dick et al., 2014; Drevytska et al., 2012). However, the role of HIFs in GDM pathogenesis remains unclear.

The aim of our study was to evaluate the correlation between *HIF3A* and GDM development. We determined mRNA level of *HIF3A* and methylation level of *HIF3A* promoter region in the omental tissue of GDM patients. Our study further investigated the mechanisms of how *HIF3A* was regulated by E2, which might be a promising target to develop diagnostic and therapeutic strategies.

## 2 MATERIALS AND METHODS

### 2.1 Clinical sample collection

Our experiments were performed by double‐blinded way. Clinical samples were collected from 20 women with GDM or 20 healthy pregnancies. Diagnostic criteria of GDM were: fasting plasma glucose ≥100 mg/dl, 1 hr oral glucose tolerance test (OGTT) ≥ 180 mg/dl, and 2 hr OGTT ≥ 155 mg/dl. Patients with diseases, such as serious liver or acute heart failure were excluded. Omental adipose samples were taken during surgical intervention approximately from the same inferior area of big omentum in all patients, and were either immediately fixed in 10% formalin for further paraffin embedding and immunohistochemistry assay, or immediately frozen in liquid nitrogen for further analysis. Written consent was derived from all the participants. This study was approved by The Affiliated Yantai Yuhuangding Hospital of Qingdao University.

### 2.2 RNA extraction and qPCR

Total RNA was isolated with the RNeasy kit (Qiagen, Germantown, MD, USA), and cDNA was synthesized with SuperScript RT III (Invitrogen, Carlsbad, CA, USA). The mRNA levels were measured by real‐time PCR performed in SYBR Green I on 7900 real‐time PCR detection system (Bio‐Rad, Hercules, CA, USA). Primer sequences were listed as follows: *HIF1A* (forward: 5′‐GAAAGCGCAAGTCTTCAAAG‐3′; reverse: 5′‐TGGGTAGGAGATGGAGATGC‐3′); *HIF2A* (forward: 5′‐CAGGCAGTATGCCTGGCTAATTCCAGTT‐3′; reverse: 5′‐CTTCTTCCATCATCTGGGATCTGGGACT‐3′); *HIF3A* (forward: 5′‐GTCGGAGAGTATCGTCTGTGTC‐3′; reverse: 5′‐TCTGCGAGAGTGTTGCTCCGTT‐3′); estrogen receptor α/estrogen receptor 1 (*ESR1*, OMIM association number, 133,430) (forward: 5′‐GCTACGAAGTGGGAATGATGAAAG‐3′; reverse: 5′‐TCTGGCGCTTGTGTTTCAAC‐3′); solute carrier family 2 member 4 (*SLC2A4*, OMIM association number, 138,190) (forward: 5′‐CAAAGCATCGACCAGTGCTA‐3′; reverse: 5′‐TGGACAGCACTGACTTCCAG‐3′); *GAPDH *(OMIM association number, 138,400) (forward: 5′‐TGTGTCCGTCGTGGATCTGA‐3′; reverse: 5′‐CCTGCTTCACCACCTTCTTGA‐3′). PCR was carried out for 35 cycles using the following conditions: denaturation at 95°C for 20 s, annealing at 58°C for 20 s, and elongation at 72°C for 20 s. Tubulin was used as internal control.

### 2.3 Serum estradiol detection

Serum specimens from GDM patients and healthy women were collected at the time of cesarean delivery. The level of estradiol (E2) was detected via Modular E170 platform electrochemiluminescence immunoassays (Roche Diagnostics, Indianapolis, IN, USA) within 30 min.

### 2.4 CpG methylation detection

CpG islands on the *HIF3A* promoter region and primers for bisulfite sequencing PCR (BSP) were predicted via “methprimer”. DNA fragments were amplified using primers (forward: 5′‐TGGTTGAAGGGTTATTTAGGG‐3′; reverse 5′‐ACTCTATCCCACCCCTTTT‐3′). Genomic DNA was treated via Methylamp DNA Modification Kit (Epigentek, Farmingdale, NY, USA), DNA was amplified using EpiTaqHS kit (Takara, Dalian, China). All experiments were performed according to the manufacturer's instruction (Huang et al., 2018).

### 2.5 Cell culture

For in vitro experiment, mouse 3T3‐L1 adipocytes were ordered from American Type Culture Collection. 3T3‐L1 adipocytes (5 × 10^4^) were cultured and treated with different concentrations of E2. All the experiments were repeated three times.

### 2.6 Statistical analysis

All *p*‐values were carried out using the SPSS software. All error bars represented mean ± SEM derived from three independent experiments. Statistical significance was determined by one way ANOVA. In all cases, *p* < 0.05 were considered to be statistically significant.

## 3 RESULTS

### 3.1 Baseline clinical characteristics of GDM patients compared with healthy controls

Baseline clinical characteristics of 20 GDM patients and 20 healthy controls were showed in Table 1. Based on our clinical information, we found that body mass index (BMI), systolic blood pressure, diastolic blood pressure, and hemoglobin (HbA1c) had no significant differences between the two groups (Table 1). Furthermore, the glucose levels during the oral glucose tolerance test were significantly increased in patients with GDM (*p* < 0.001) (Table 1). In addition, fasting insulin level was also dramatically increased in patients with GDM compared with control group (*p < *0.05) (Table 1).

**Table 1:** Clinical characteristics of the participants in this study

	Control	Gestational diabetes mellitus	*p* Value
*n*	20	20	
Age (years)	27.5 (3.5)	28 (2.25)	0.478
Gestational age at delivery (days)	275 (7.25)	274 (7.5)	0.734
Birthweight (g)	3,392.5 (433.75)	3,487.5 (438.75)	0.607
BMI (kg/m^2^) before pregnancy	24 (4.075)	24.25 (5.6)	0.337
Systolic blood pressure (mmHg)	120 (14.5)	119 (22.25)	0.473
Diastolic blood pressure (mmHg)	76.5 (8.5)	74.5 (6.5)	0.578
HbA1c (%)	5.35 (0.55)	5.4 (0.275)	0.87
Glucose 0 hr (mmol/l)	4.3 (0.25)	4.7 (0.425)	<0.001*
Glucose 1 hr (mmol/l)	7.5 (0.55)	10.75 (1.75)	<0.001*
Glucose 2 hr (mmol/l)	6.5 (0.325)	8.6 (1.15)	<0.001*
Fasting insulin (pmol/l)	57.9 (36.65)	81.45 (36.525)	0.044*

Note

Values were shown as median (interquartile range).

* *p* < 0.05 as assessed by Mann–Whitney *U*‐test.

### 3.2 *HIF3A* was downregulated in the omental tissue from GDM patients

**Figure 1:**
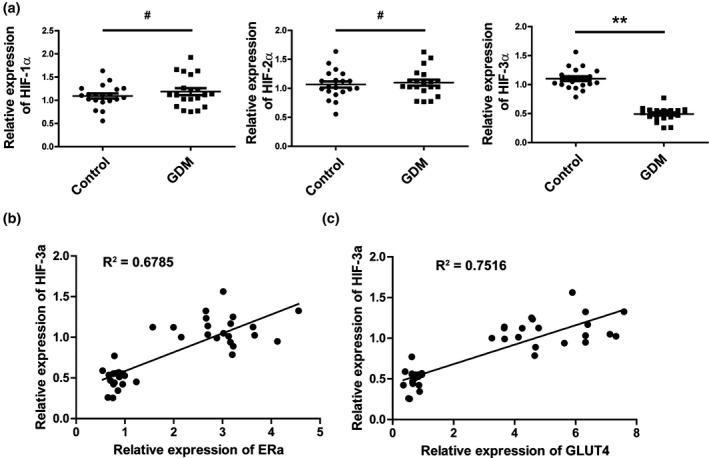
*HIF3A* was down‐regulated in the omental tissue from gestational diabetes mellitus patients. (a) Representative scatter plots showing the expression level of *HIF1A*, *HIF2A,* and *HIF3A* in the omental tissue from gestational diabetes mellitus patients and healthy controls. ^#^ *p* > 0.05, **p < 0.01. (b) Spearman's correlation analysis of ***ESR1*** and ***HIF3A*** expression levels (*R* ^2^ = 0.6785, *p < *0.001). (c) Spearman's correlation analysis of ***SLC2A4*** and ***HIF3A*** protein levels (*R* ^2^ = 0.7516, *p* < 0.001)

To further investigate whether HIF family proteins were involved in the regulation of GDM development, we analyzed the expression levels of ***HIF1A***, ***HIF2A,*** and ***HIF3A*** in the omental tissue from GDM patients and healthy controls. The results showed that the expression level of ***HIF3A*** (0.493 ± 0.115), but not ***HIF1A*** (1.187 ± 0.327) or ***HIF2A*** (1.097 ± 0.228), was significantly decreased in omental tissues from GDM patients (Figure 1a), which indicated that ***HIF3A*** expression level might have a negative correlation with GDM development. We next detected the correlation between the expression level of ***HIF3A*** and *ESR1*. We found that the expression level of ***HIF3A*** was correlated with *ESR1* (Figure 1b). Furthermore, *SLC2A4*, an insulin‐regulated glucose transporter, was also correlated with *HIF3A* (Figure 1c). These above results indicated that *HIF3A* expression level was downregulated and positive correlated with both *ESR1* and *SLC2A4* in GDM patients.

### 3.3 Serum estradiol levels were upregulated in GDM patients

**Figure 2:**
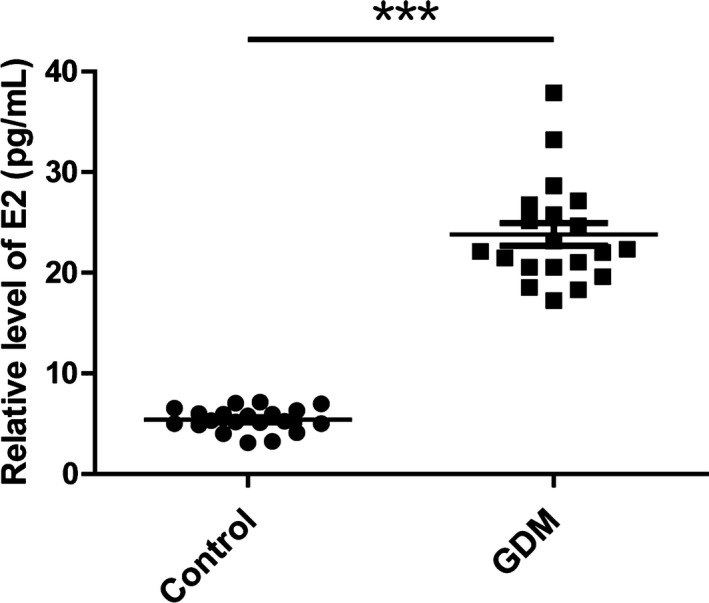
Serum estradiol levels were up‐regulated in gestational diabetes mellitus Patients. The serum level of estradiol was determined by electrochemiluminescence. ***p < 0.001

Estradiol level has been reported to be upregulated in GDM patients. Thus, we tested the E2 level in the serum samples from GDM patients and controls. Consistently, we found that the level of E2 was significantly higher in GDM patient serum (23.068 ± 3.158 pg/ml) than in the control group (5.008 ± 1.239 pg/ml) (Figure 2).

### 3.4 The CpG islands in *HIF3A* promoter were highly methylated in GDM patients

**Figure 3:**
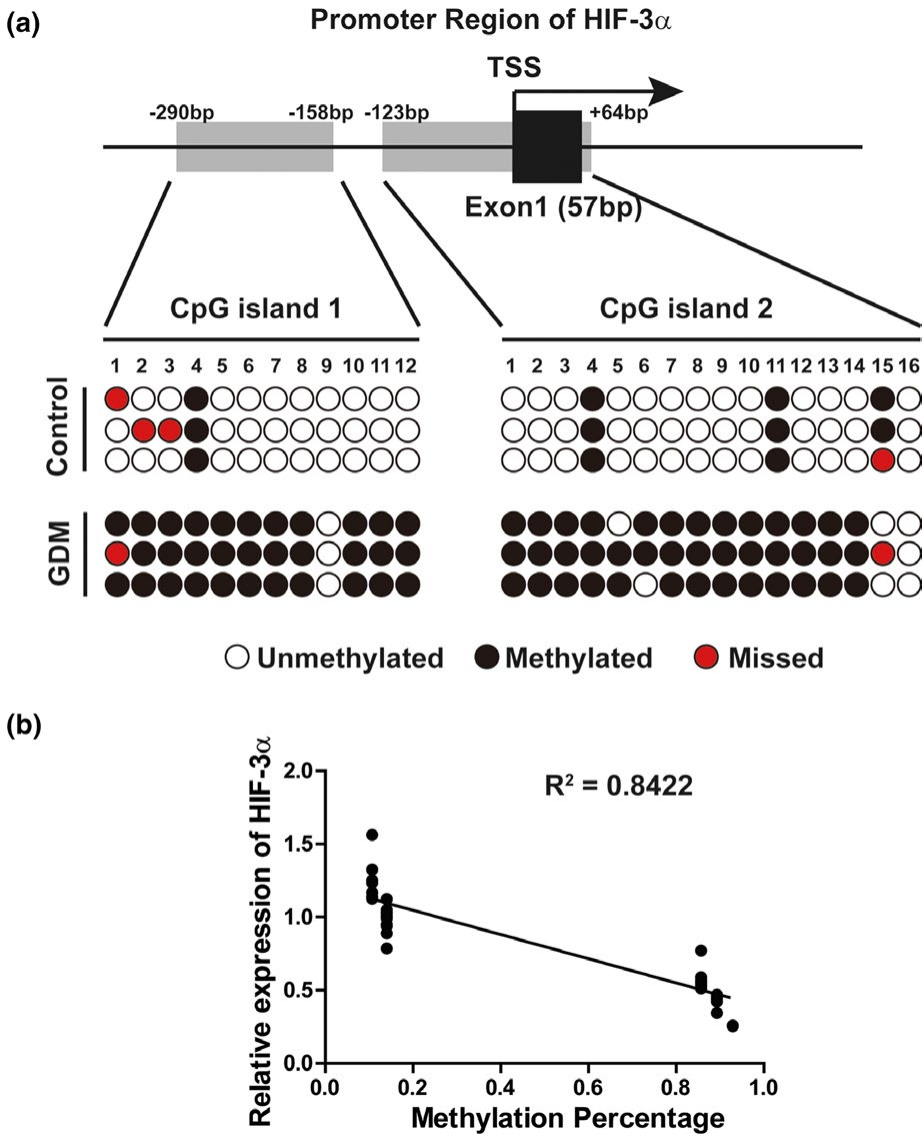
*HIF3A* promoter CpG islands were highly methylated in gestational diabetes mellitus patients. (a) BSP analysis of CpG islands methylation status in ***HIF3A*** promoter region: Two CpG islands, from −290 to −158 (CpG island 1) and from −123 to +64 (CpG island 2) in ***HIF3A*** promoter region (the transcriptional start site was set as +1), were predicted via online software “methprimer” (Top); BSP analysis of the promoter CpG islands methylation status in the omental tissue from healthy control or gestational diabetes mellitus patients were shown in the bottom. (b) Pearson correlation analysis of ***HIF3A*** expression and CpG islands methylation degree; ***HIF3A*** mRNA level was significantly negatively correlated with the CpG islands methylation degree (*R* ^2^ = 0.8422, *p* < 0.001)

To address whether the decreased expression of ***HIF3A*** in GDM patients was due to difference in methylation status, we predicted two CpG islands in the promoter region of ***HIF3A*** and designed the primers through “methprimer” software. We next compared the methylation level of those two predicted CpG islands between GDM patients and control groups. The results showed that the methylation level was significantly higher in CpG islands of GDM patients than control groups (Figure 3a). Furthermore, we analyzed correlation between ***HIF3A*** expression and CpG island methylation degree, and found a strong negatively correlation (Figure 3b). The above results suggested that the CpG islands of ***HIF3A*** promoter were highly methylated in GDM patients, resulting in a low expression level of ***HIF3A***.

### 3.5 The CpG islands of *HIF3A* promoter were highly methylated under E2 treatment

**Figure 4:**
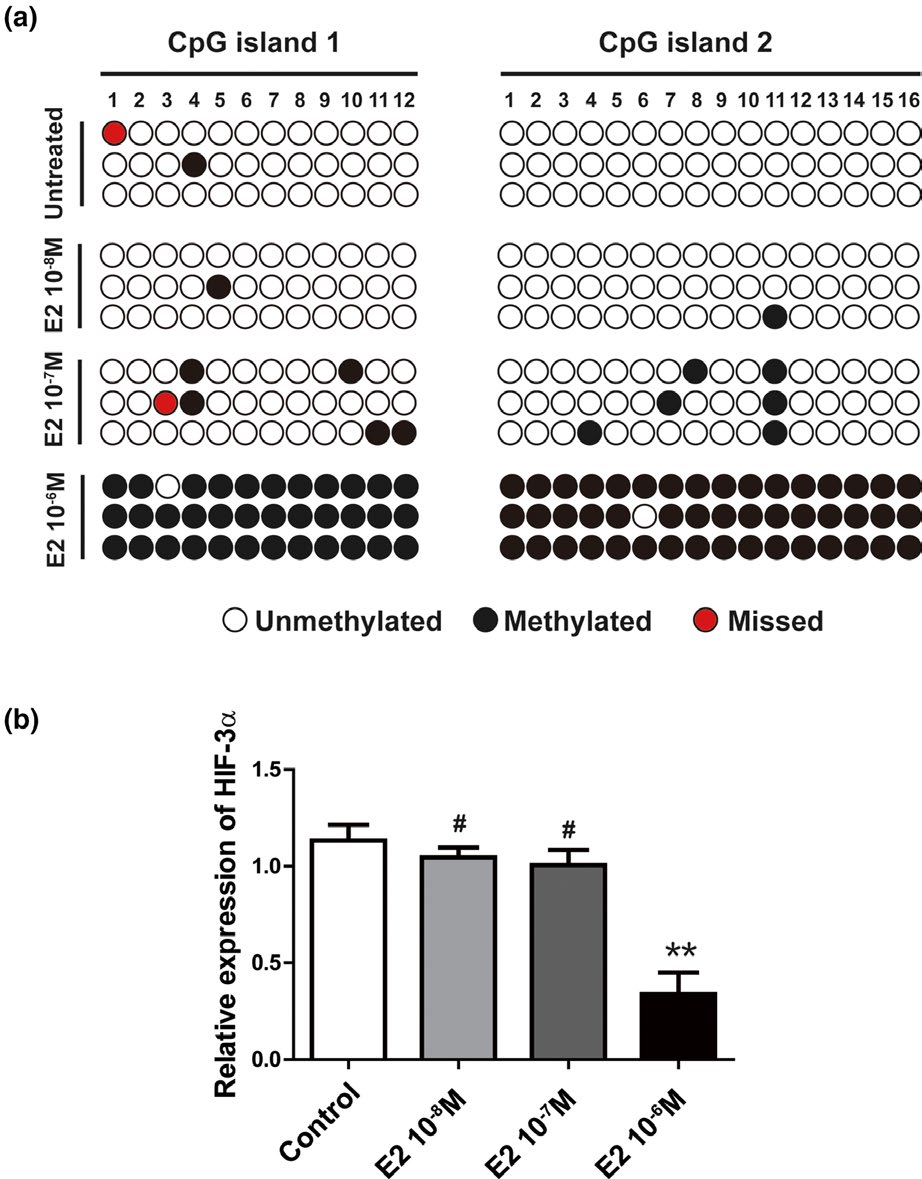
E2 treatment could highly methylated *HIF3A* promoter CpG islands in 3T3‐L1 adipocytes. (a) 3T3‐L1 adipocytes were treated with different concentration of E2 as indicated for 48 hr and the BSP analysis was used to determine the methylation status of the promoter CpG islands; (b) Q‐PCR showed the relative mRNA level of ***HIF3A*** in 3T3‐L1 adipocytes with the different treatments. Data were represented as mean ± *SEM*. *n* = 3 independent experiments. ^#^ *p* > 0.05, **p < 0.01

Considering that E2 level was upregulated in GDM patients, we next asked whether the methylation level of ***HIF3A*** promoter region could be induced by E2 treatment. By detecting the methylation level of two predicted CpG islands in 3T3‐L1 adipocytes with or without E2 treatment, we found that both two CpG islands were highly methylated upon E2 treatment (Figure 4a). The methylation level of CpG islands was increased in an E2 dose‐dependent manner (Figure 4a). Meanwhile, we also tested the expression level of ***HIF3A*** under treatment with different concentrations of E2. Consistently, the expression of ***HIF3A*** was significantly downregulated upon E2 treatment (Figure 4b).

## 4 DISCUSSION

Epigenetic modification alters chromatin structure and DNA accessibility, which play an important modulatory role within the cell. Recent studies have showed that deregulation of DNA methylation occurs in different diseases, highlighting their critical roles in therapeutic applications (Kishida et al., 2001; Miura et al., 2001). Although many efforts have been made to understand the molecular and cellular mechanisms of GDM development, the role and significance of DNA methylation in GDM remain incompletely understood. Our data provide evidences suggesting that the expression of ***HIF3A*** is down‐regulated in omental tissues of GDM patients (Figure 1a). Further study showed that the methylation of CpG island within ***HIF3A*** promoter was significantly increased in GDM patients (Figure 3), leading to ***HIF3A*** gene silencing. Our results indicate an important role of ***HIF3A*** in GDM development, making it a potential candidate for therapeutic applications.

Consistently, our study is supported by previous studies reporting that ***HIF3A*** was involved in adipocyte differentiation, insulin resistance, and glucose metabolism (Haertle et al., 2017), which are key factors contributing to GDM pathogenesis (Davis, Gunderson, Gunderson, Gyllenhammer, & Goran, 2013). For example, two CpG methylation sites in intron 1 of ***HIF3A*** gene, cg16672562, and cg46801562, have significant correlation with obesity (Lee et al., 2017). Practically, cg16672562 was shown to be a blood DNA methylation marker for obesity, since it was positively associated with BMI, plasma glucose level and waist‐hip ratio (Lee et al., 2017). Another genome‐wide analysis also reported that methylation of ***HIF3A*** in adipose tissue was involved in adipose tissue dysfunction (Pfeiffer et al., 2016); for instance, ***HIF3A*** methylation site cg22891070 was higher in visceral adipose tissue than subcutaneous adipose tissue, associated with hip, subcutaneous and visceral fat mass (Pfeiffer et al., 2016). Moreover, there was a positive association between BMI and ***HIF3A*** DNA promoter methylation in the blood of T2D patients (Main et al., 2016). Furthermore, ***HIF3A*** expression in subcutaneous adipose tissue was adversely associated with insulin resistance (Main et al., 2016). In addition, a methylation screening via bisulfite pyrosequencing reported that the average methylation of 11 CpG islands in ***HIF3A*** was significantly higher in the blood of GDM patients compared to the controls (Haertle et al., 2017). Taken together, our data have revealed a role of ***HIF3A*** in GDM development, and further study should focus on finding downstream target genes of ***HIF3A***.

The massive secretion of various hormones during pregnancy contributes to insulin resistance‐induced GDM (Shi et al., 2014). Our studies showed that the serum level of E2 was significantly increased in GDM patient compared to control group. The level of E2 in a healthy person usually varies from 10^−9^ to 10^−7^ M: in steady state, E2 level is about 10^−9^ M; during menstrual period, it is around 10^−8^ M; it is further upregulated to 10^−7^ M during pregnancy (Xie et al., 2011). In GDM patients, the E2 concentration has a 4–5 fold increase compared to healthy individuals (Figure 2). Further studies demonstrated high dose of E2 treatment (10^−6^ M) induced methylation of ***HIF3A*** in 3T3‐L1 adipocytes, whereas low concentration (10^−8^ to 10^−7^ M) of E2 did not influence ***HIF3A*** methylation. These results suggested the excessive production of E2 might be an important contributor to ***HIF3A*** methylation in GDM patients. Consistent with our results, unconjugated E2 levels were significantly associated with increased risk for developing GDM (Hur et al., 2017), which has a potent antagonistic activity when present with E2. These results suggest E2 might have the potential to become a serum biomarker to predict GDM development. Nevertheless, results of our study were obtained from a relatively small sample size, and should be verified in further multi‐centered studies employing larger samples.

## 5 CONCLUSIONS

In summary, our findings indicate an unrecognized role of ***HIF3A*** methylation in GDM, and suggest it as a promising target for GDM treatment. In vitro study has showed that the methylation of ***HIF3A*** CpG island is upregulated by E2. Moreover, the expression of ***HIF3A*** is positively associated with ***ESR1*** and ***SLC2A4***. These studies may help us better understand the role of DNA methylation in GDM, opening a new door for more targeted therapies.

## CONFLICT OF INTEREST

The authors declare that they have no conflict of interest.
